# Material Removal and Surface Integrity Analysis of Ti6Al4V Alloy after Polishing by Flexible Tools with Different Rigidity

**DOI:** 10.3390/ma15051642

**Published:** 2022-02-22

**Authors:** Xiaolong Ke, Wei Wu, Chunjin Wang, Yongheng Yu, Bo Zhong, Zhenzhong Wang, Tianyi Wang, Jianji Fu, Jiang Guo

**Affiliations:** 1 School of Mechanical and Automotive Engineering, Xiamen University of Technology, Xiamen 361024, China; kexiaolong@xmut.edu.cn (X.K.); wuwei@s.xmut.edu.cn (W.W.); yonghengyu0015@163.com (Y.Y.); georgexmu@139.com (J.F.); 2 State Key Laboratory of Ultra-Precision Machining Technology, Department of Industrial and Systems Engineering, The Hong Kong Polytechnic University, Hung Hom, Kowloon, Hong Kong, China; 3 Department of Automation, Tsinghua University, Beijing 100084, China; zhongbo_foerc@163.com; 4 School of Aeronautics and Astronautics, Xiamen University, Xiamen 361005, China; wangzhenzhong@xmu.edu.cn; 5 National Synchrotron Light Source II (NSLS-II), Brookhaven National Laboratory, Upton, NY 11973, USA; tianyi@bnl.gov; 6 Key Laboratory for Precision and Non-Traditional Machining Technology of Ministry of Education, Dalian University of Technology, Dalian 116024, China; guojiang@dlut.edu.cn

**Keywords:** Ti6Al4V, polishing, material removal rate, surface integrity, ultra-precision machining

## Abstract

Ti6Al4V alloy has been widely used in many fields, such as aerospace and medicine, due to its excellent biocompatibility and mechanical properties. Most high-performance components made of Ti6Al4V alloy usually need to be polished to produce their specific functional requirements. However, due to the material properties of Ti6Al4V, its polishing process still requires significant development. Therefore, this study aimed to investigate the performance of polishing Ti6Al4V by using tools with different rigidities. Two kinds of bonnet tool were used, namely a pure rubber (PR) bonnet and a semirigid (SR) bonnet. The characterization of material removal and surface integrity after polishing was conducted through a series of experiments on a 6-DOF robotic polishing device. The results demonstrate that both bonnet tools successfully produce nanometric level surface roughness. Moreover, the material removal rate of the SR bonnet tool is significantly higher than that of the PR bonnet, which is consistent with the material removal characteristics of glass polishing in previous research. In addition, the presented analysis on key polishing parameters and surface integrity lays the theoretical foundation for the polishing process of titanium alloy in different application fields.

## 1. Introduction

Ti6Al4V alloy is an α+β-type, dual-phase alloy with excellent material properties [1,2,3], including low density, high mechanical strength, good corrosion resistance, high biocompatibility, and other distinct mechanical and physical properties. It also offers the ability to adjust these material properties to a large extent by optimizing its microstructure and surface properties [4,5,6,7]. It has been widely used in the manufacture of turbine engine blades, compressor discs, jet engines in the aerospace industry, and armor steel used for bullet-proofing in the military industry [8,9,10]. Furthermore, Ti6Al4V is commonly used in the biomedical field due to its high biocompatibility [11,12], such as artificial hip joints, compression hip screws, dental implants body, bone plate, heart catheter, artificial heart valve for orthodontic surgery, etc. [13,14,15,16,17]. Furthermore, low surface roughness is usually required to obtain superior functionality for most of its applications, such as implants and turbine blades [18,19]. However, it is a typical hard-to-machine material like other multi-phase alloys [19,20,21,22,23] which is difficult to polish. Therefore, it is urgently necessary to find an efficient Ti6Al4V alloy polishing method to improve the overall surface quality.

Currently, titanium alloy parts (such as aerospace engine blades) are mainly polished using time-consuming and labor-intensive artificial methods. The surface quality is thus unstable, which restricts its practical applications. Many researchers have attempted to grind and polish titanium alloys using CNC machine tools or robot-assisted devices. Axinte et al. [24] proposed to use belts as the final finishing method of Ti6Al4V heat-resistant alloy parts to further improve the surface quality. Perry et al. [25] studied the method of pulse laser micro-polishing (PLµP) for reducing the surface roughness of micro-milled Ti6Al4V specimens. The experiment proved that PLµP is an appropriate process that can improve the surface roughness of micro-milled Ti6Al4V. Li et al. [26] proposed a novel constrained abrasive flow polishing technology. They introduced a triangular constraining plate to perform abrasive flow polishing on the surface of complex titanium alloys, which effectively improved the dimensional accuracy and surface quality. Smith et al. [27] proposed precision diamond micro-arrays for Ti6Al4V grinding, which achieved a 3.5 times improvement in the surface finish and a 21.5 times improvement in the flatness of Ti6Al4V workpieces compared with the conventional method. Zhang et al. [28] developed a new chemical mechanical polishing method and slurry to further improve polishing performance for titanium alloys. Li et al. [29] studied the electrochemical grinding (ECMG) process for titanium alloy, which can provide high-efficiency and precise machining of titanium alloy integral structural parts. Xiao et al. [30] investigated the belt high-efficiency finishing (BEF) method to establish a material removal model to achieve the final precision-finishing of titanium alloy parts. Erdoğan et al. [31] demonstrated the use of short-pulse fiber lasers to process Ti6Al4V titanium alloy dental implants with excellent uniformity and repeatability. Lou et al. [32] attempted to apply electric pulse treatment (EPT) to improve the ultra-precision machining performance of titanium alloys. The results indicated that a better surface finish of titanium alloys was achieved after EPT.

Even though the polishing methods mentioned above have been successfully applied to the polishing of Ti6Al4V alloy, the polishing cost is generally high, and the process is not stable enough for mass production. Further in-depth research and development on the polishing technique of titanium alloys is still necessary.

A bonnet polishing technology adopting the precession movement (called “bonnet polishing” for brief) was developed by Zeeko Ltd. (Leicestershire, United Kingdom) in collaboration with the Optical Science Laboratory at University College London and Loh Optik maschinen [33]. This technology uses a rotating inflated spherical membrane tool (the “bonnet”) that naturally molds itself to the local aspheric surface and maintains stability to provide natural smoothing. Compared to artificial polishing processes, it shows higher removal efficiency, excellent removal of mid-spatial frequencies, and the ability to control the edge form [34,35]. Walker et al. [36], Beaucamp et al. [37,38], and Su et al. [39] used bonnet tools to polish optical lenses, such as aspherical lenses, molds, freeform surfaces, and structured surfaces, achieving satisfactory surface quality. However, the removal rate of the used bonnet tools was low, so the overall process was still inefficient.

To improve removal efficiency, in our previous research, we proposed a semirigid (SR) bonnet tool [40,41] that we successfully applied to high-efficiency, large-aperture optics polishing. Compared to the traditional pure rubber (PR) bonnet tool, the SR bonnet greatly enhances the material removal rate while maintaining a certain degree of flexibility [40,41,42]. Therefore, in this paper, we further investigate the feasibility of applying SR bonnet tools for the polishing of Ti6Al4V alloys. With the polishing experiment using the PR bonnet tool performed as a reference, the performances of the two kinds of bonnet tool in Ti6Al4V polishing were carefully studied. Finally, key polishing parameters and the surface integrity before and after polishing were analyzed. The conclusion to this study can be potentially used as a theoretical guide to the polishing process of Ti6Al4V in different application fields.

## 2. Experiment

### 2.1. Material

The material of the workpiece used in this study was α+β type, two-phase Ti6Al4V alloy (BAOTi in China) with a density of 4.51 g/cm^3^ and a hardness of 31HRC, formed by a rolling process. In addition, the titanium alloy material used in this experiment underwent annealing heat treatment before leaving the factory. The chemical composition complies with the GB/T3624-2007 standard, and its basic composition is shown in Table 1.

**Figure 1:**
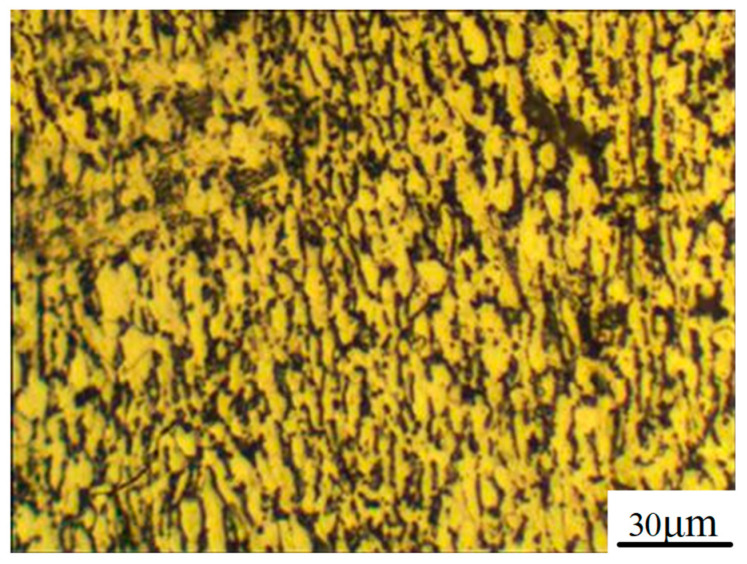
Metallographic structure of Ti6Al4V.

Figure 1 shows the metallographic structure observed by a metallographic microscope. As the titanium alloy formed by saw blade cutting had many scratches on its surface and the flatness was poor, it could not be directly polished. To ensure the unity of the experiment, a titanium alloy sheet with length × width × thickness = 100 mm × 100 mm × 10 mm was used as the sample for the mechanical characteristics experiment. The surface of the sample was ground by a 150-mesh grinding wheel to 3~4 λ (λ = 632.8 nm) peak-to-valley (PV), together with a surface average arithmetic roughness (Sa) of 0.2~0.3 μm.

Alumina polishing slurry with different grades of size was used as the polishing slurry. The samples were ultrasonically cleaned in alcohol for 10 min and dried on a clean and ventilated laboratory bench before and after polishing.

### 2.2. Experimental Setup

**Figure 2:**
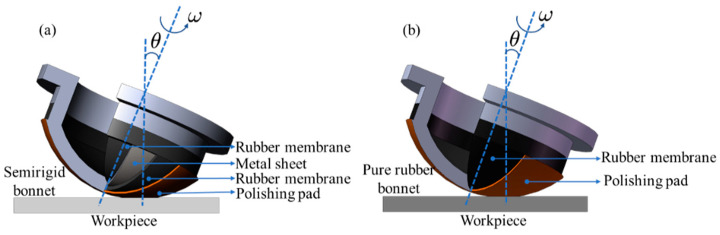
Structure of (**a**) the semirigid (SR) bonnet and (**b**) pure rubber (PR) bonnet.

The SR bonnet and PR bonnet with radii of 40 mm were used in the experiment, as shown in Figure 2. The structure of SR bonnet is shown in Figure 2a [41]. It is composed of three layers. The outermost and innermost layers are rubber membranes, and the middle layer is a stainless-steel metal sheet with a thickness of 0.3 mm, which helps increase the tool rigidity and flexibility. The outermost rubber film is covered with a polishing pad. The flexible layer of the PR bonnet shown in Figure 2b is made of rubber with a shore hardness of 80.

**Figure 3:**
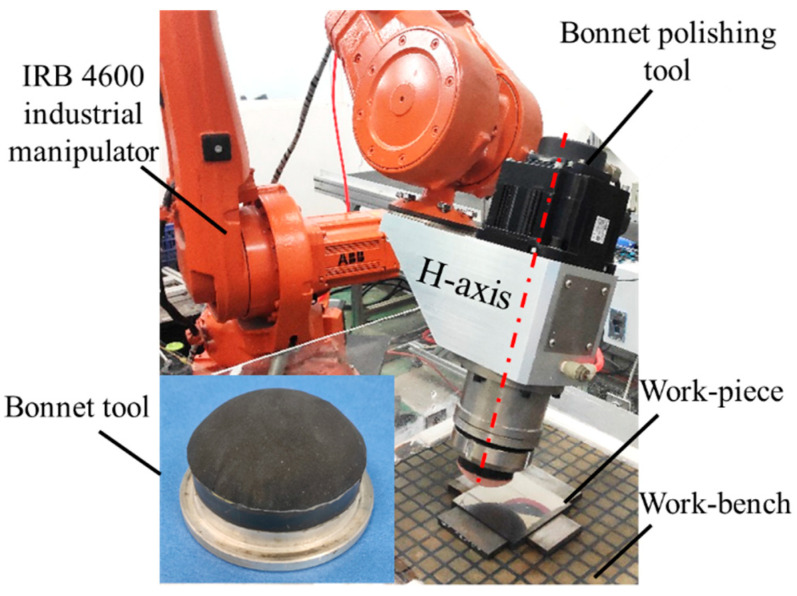
Robot-assisted bonnet polishing system used in this study.

When polishing with the SR bonnet or PR bonnet, the bonnet tool contacts the surface to be polished, squeezing the polishing abrasive. In the meantime, the rotation of the bonnet tool drives the movement of the abrasive to continuously scrape the surface, forming a small amount of material to remove. Figure 3 shows the self-developed, robot-assisted polishing device that was used in the experiment, which is equipped with a 6-DOF ABB robot arm (IRB4600-60/2.05). This 6-DOF industrial robot arm includes three rotation axes and three swing axes that can handle a payload of up to 60 kg. The farthest achievable distance of the arm is 2.05 m, and the repeated positioning accuracy is ±0.05 mm. The bonnet tool was fixed at the end of the H-axis, and the rotation of the bonnet tool was controlled through a PLC electric control cabinet.

The polishing pad used in this experiment was UN1NAP5-40W damping cloth (Universal Photonics, Central Islip, NY, USA). It is a velvet-like polishing material with fine texture, soft surface, porous, elastic, and long service life. It can effectively impregnate the polishing liquid during polishing to improve the removal efficiency while avoiding scratches on the workpiece. It is a high-performance, optical-level processing material [43].

### 2.3. Experimental Design

#### 2.3.1. Comparison of Material Removal Characteristics

**Figure 4:**
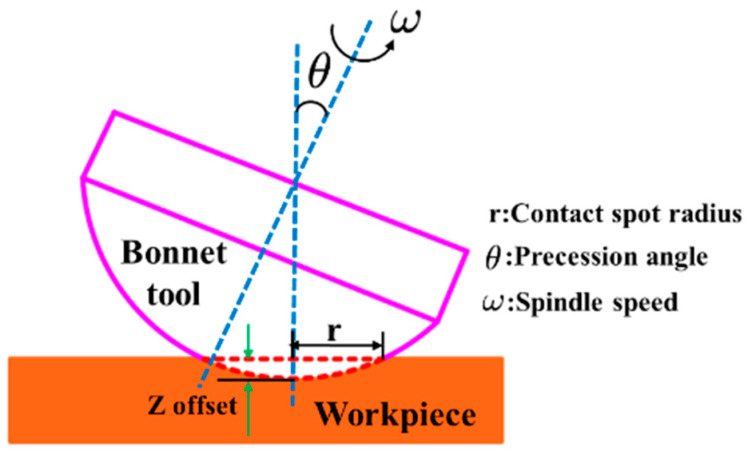
Schematic of bonnet tool polishing model.

Figure 4 shows the geometric model of bonnet tool polishing. To observe the material removal characteristics, two groups of experiments were conducted to extract the tool influence functions (TIFs) of the SR and PR bonnet tools. Following the conditions in Table 2, the first group was obtained under the same polishing conditions except for dwell time, while different tool offsets were used in the other group. The polishing slurry was ~3 wt.% alumina. The average diameter of the abrasive grain size was ~5 μm. Finally, the SR bonnet and PR bonnet tools each obtained ten TIFs.

#### 2.3.2. Uniform Polishing Experiment

To compare and analyze the polishing characteristics of the SR and PR bonnets, a uniform polishing experiment using the extracted TIFs was conducted. The experiment was performed on a Ti6Al4V alloy sample with a size of 100 mm × 100 mm × 10 mm. The sample was firstly ground to produce a form error lower than 6 μm Sa. To avoid damaging the bonnet tool and discard the influence of the edge effect, the actual polishing area was 80 mm × 80 mm, and a raster tool path with a 1 mm scanning interval was used. The other experimental parameters are detailed in Table 3. In the experiment, ten polishing cycles were performed using the SR bonnet, followed by nine cycles with the PR bonnet on the same workpiece. The surface roughness of each cycle was measured and recorded.

### 2.4. Measurement Methods

The polished surface morphology, surface energy spectrum analysis, surface chemical composition, surface roughness (Sa, Sq, and Sz), and material volume removal rate (VRR) were measured in this study to evaluate the polishing performance of the two kinds of bonnet tools on Ti6Al4V alloy. The surface morphology was measured by a Scan Electron Microscope (SEM, ZEISS sigma 500, Oberkochen, Germany) and a super depth of field 3D Microscope System (KEYENCE, VHX-2000C, Osaka, Japan). The SEM detection parameters were set at 15 kV acceleration voltage and 8.8 mm working distance. In the meantime, the surface chemical composition was analyzed through the energy-dispersive X-ray (EDX) mounted on the SEM. The crystallographic structure of the titanium alloy before and after polishing was analyzed through X-ray diffraction (XRD, Rigaku SmartLab SE, Tokyo, Japan) with the Bragg–Brentano method. A ZYGO NewView^TM^ 9000 was used to measure the TIF and surface roughness. A 10-magnification objective lens and a 0.5-magnification eyepiece were used to detect the three-dimensional shape of the entire TIF, and a 50-magnification objective lens and a 2-magnification eyepiece were used in to measure the surface roughness. The experiment data were saved in a text format, and the data points in the text were extracted into a three-dimensional matrix to calculate the volume removal rate (VRR) of TIF by performing volumetric integration.

## 3. Results and Discussions

### 3.1. Material Removal Rate Analysis

**Figure 5:**
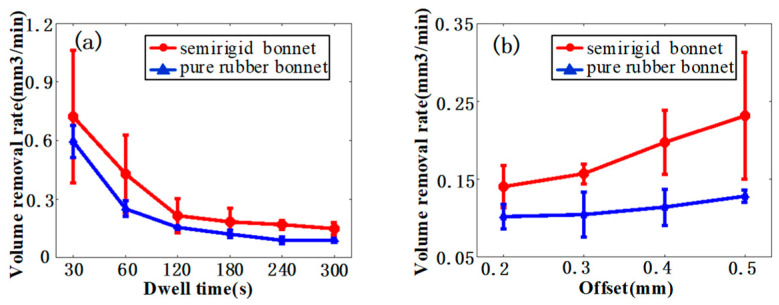
(**a**) Volume removal rate of TIFs under different dwell time, and (**b**) volume removal rate of TIFs under different offset.

The VRR’s variation with respect to the dwell time and offset of the two bonnet tools is demonstrated in Figure 5. It was found that the SR bonnet has a higher VRR than the PR bonnet under the same polishing conditions, which coincides with the results of polishing BK7 optical glasses reported in [41,44]. However, it was noted that the VRR increment is not proportional to the increase of the dwell time, as shown in Figure 5a. The VRRs of both of the two bonnet tools gradually decrease with the increase in dwell time and converge to a constant value, which is quite different from the results reported in [41,44]. When the dwell time is short, the peaks on the surface are easier to effectively remove or smoothen, leading to a high material removal rate. With the longer dwell time due to the strong tenacity of titanium alloy materials, the difficulty of material removal increases. Both variation trends between VRRs and tool offsets increase gradually.

**Figure 6:**
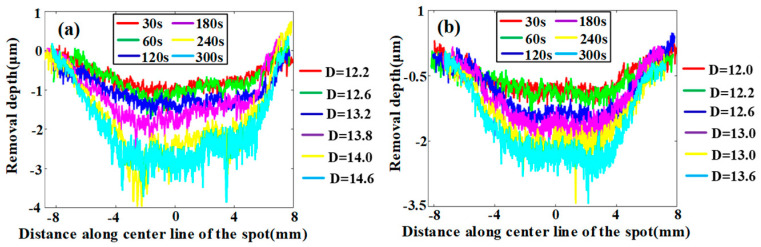
Section size of polishing point of (**a**) SR bonnet and (**b**) PR bonnet (D signifies the diameter of the spot).

**Figure 7:**
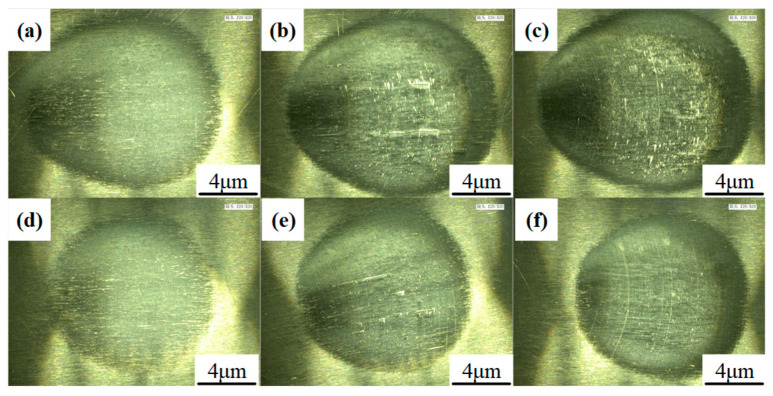
The super depth of field morphology of TIFs subjected to different bonnet tools: (**a**–**c**) The TIFs of the SR bonnet under different dwell times of 30 s, 180 s, and 300 s; (**d**–**f**) the TIFs of PR bonnet under different dwell times of 30 s, 180 s, and 300 s.

Similarly to previous VRR-offset investigation experiments on BK7 glass [41,44], a linear relationship was found between the VRR and the tool offset. Table 4 shows the dimensions of the cross-sectional profiles of the TIFs of the SR bonnet and the PR bonnet under different dwell times, and Figure 6 presents the corresponding measured cross-sectional profiles of the SR bonnet and PR bonnet. Figure 7a–f show the super depth of field morphology of the SR bonnet and the PR bonnet under different dwell times. It can be seen from all these comparisons that under the same conditions, the radius and depth of the cross-section of the SR bonnet polishing point are larger than those of the PR bonnet, which also indicates that increases in bonnet hardness lead to greater material removal rates.

### 3.2. Surface Roughness Analysis inside the TIF Region

**Figure 8:**
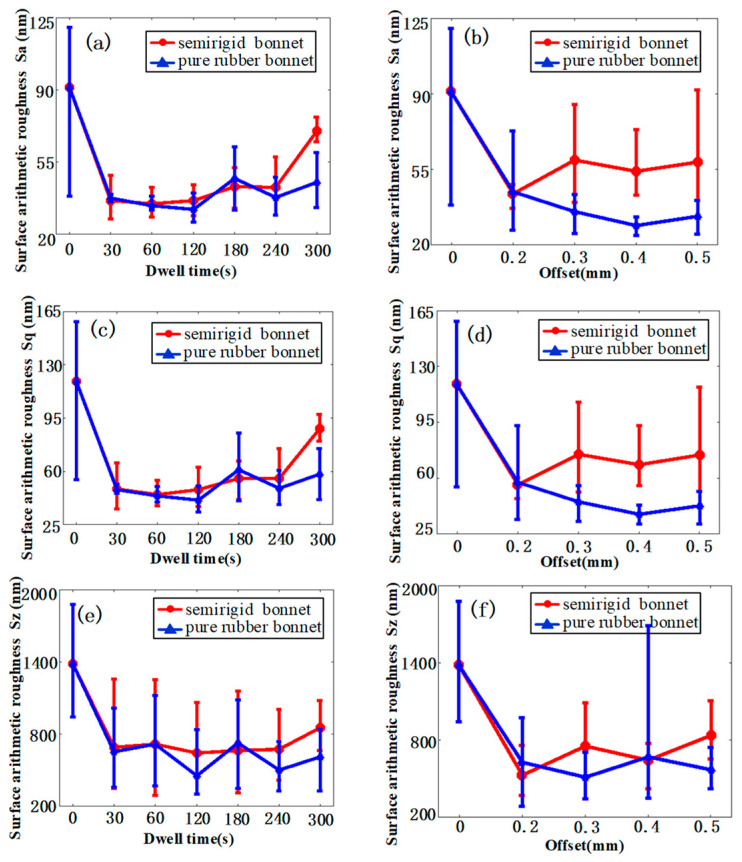
Surface roughness in Sa of the polishing spot of different bonnet tools under (**a**) different dwell times, and (**b**) different offsets by different bonnet tools. Surface roughness in Sq of the polishing spot of different bonnet tools under (**c**) different dwell times, and (**d**) different offsets by different bonnet tools. Surface roughness in Sz of the polishing spot of different bonnet tools under (**e**) different dwell times, and (**f**) different offsets by different bonnet tools.

Figure 8a,c,e demonstrate the relationship between the roughness (in Sa, Sq, and Sz) and dwell time in the center of the TIF spot. The results show that both tools can greatly reduce the surface roughness in a short time. After polishing for 30 s, Sa can be reduced from 91 nm to ~36 nm. With the increase in time, the surface roughness converges to a value and the further increment is tiny. As shown in Figure 8b,d,f, the SR bonnet achieves the lowest surface roughness at the tool offset of 0.2 mm, while it is 0.4 mm for the PR bonnet. This is because a larger tool offset means a higher contact pressure, and the contact pressure of the SR bonnet is much larger than the PR bonnet. Furthermore, we found that under the same dwell time, the surface roughness achieved by the PR bonnet is slightly less than that obtained with the SR bonnet, which indicates that the SR bonnet can be used for the pre-polishing of titanium alloy, after which the PR bonnet can be introduced for a finer finish.

### 3.3. Surface Integrity Analysis after Uniform Polishing

**Figure 9:**
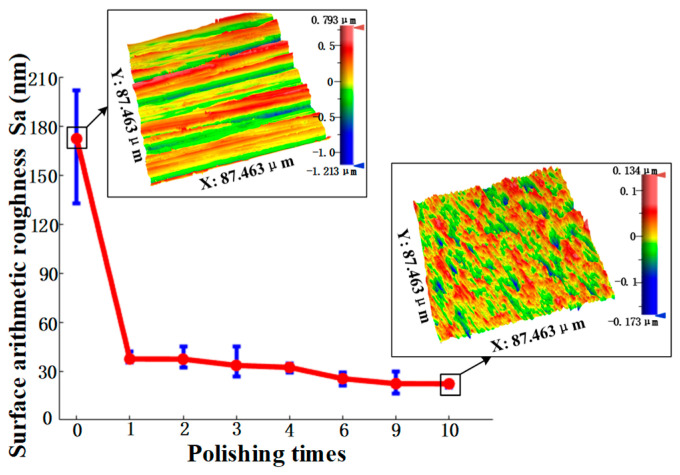
The roughness of the semirigid bonnet under different polishing times.

The uniform polishing of the Ti6Al4V surface after grinding was also conducted. Since the SR bonnet has higher VRR than the PR bonnet, the SR bonnet was used for the pre-polishing and the PR bonnet was used for the fine polishing in this experiment. Figure 9 shows the surface roughness variations of the SR-bonnet-polished surfaces under the uniform polishing experiment on Ti6Al4V alloy. The initial roughness of the Ti6Al4V alloy used in the experiment was 172.1 nm Sa, and the PV of the surface form exceeded 3 μm. The results reveal that with more polishing cycles, the surface roughness of Ti6Al4V alloy decreases gradually and converges to 22.2 nm Sa after 10 cycles. The 3D contour of the surface roughness measurement results before and after 10 cycles polishing are also demonstrated in Figure 9. It can be observed that the surface of Ti6Al4V alloy has obvious grinding tool marks and deep grooves before polishing. After 10 cycles of polishing, the surface scratches and grooves were effectively removed, which indicates that the SR bonnet is feasible for pre-polishing the surface of titanium alloy after grinding.

**Figure 10:**
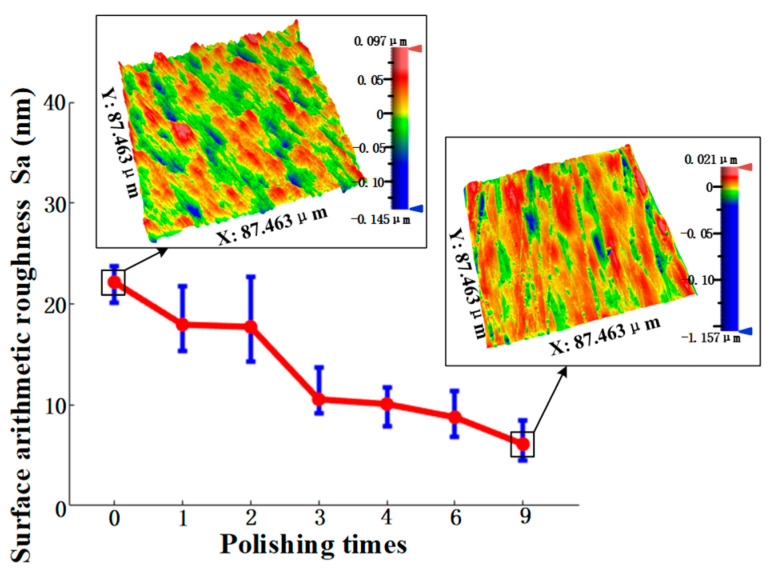
Pure rubber bonnet roughness under different polishing times.

**Figure 11:**
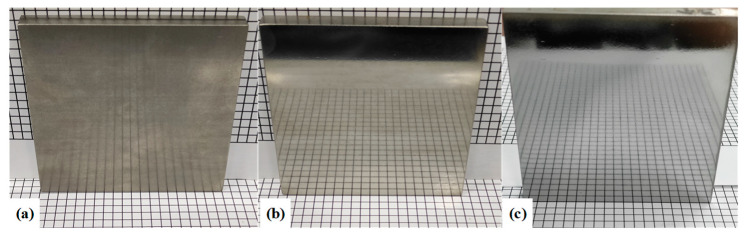
Comparison of titanium alloy surface effects before and after polishing with SR bonnet and PR bonnet: (**a**) Before polishing, (**b**) uniform polishing after SR bonnet, and (**c**) uniform polishing after PR bonnet.

According to the above results, the use of the SR bonnet to polish the above titanium alloy samples reached the convergence value of surface roughness. Hence, finer polishing was subsequently conducted using the PR bonnet. Figure 10 shows the measured surface roughness after each polishing cycle using the PR bonnet. It can be seen that the surface roughness of the Ti6Al4V titanium alloy was further reduced to 17 nm Sa after 2 cycles of polishing. Polishing slurry with a smaller abrasive size of 0.5 μm was used for the next two cycles of polishing, and the surface roughness of 10 nm Sa was then obtained. Finally, a polishing liquid with an average particle size of 0.05 μm was used for further improvement of the surface. After five cycles of polishing, the surface roughness of the Ti6Al4V alloy converged to 6.1 nm Sa. Figure 11 shows the comparison of the surface of titanium alloy before and after polishing with the SR bonnet and PR bonnet, respectively; a highly smooth, mirror-like surface was successfully produced.

### 3.4. Surface Topography Analysis of Ti6Al4V before and after Polishing

**Figure 12:**
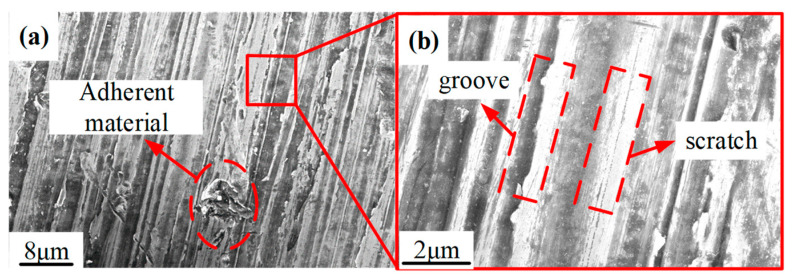
SEM photograph of Ti-6Al-4V alloy after grinding: (**a**) Adherent material on titanium alloy surface after grinding; (**b**) plastic grooves and scratches on the surface of titanium alloy after grinding.

The surface micro-topography of the Ti6Al4V surface before polishing is shown in Figure 12. It can be seen that, due to the extrusion of the abrasive grains of the grinding wheel, plastic grooves and a large number of scratches formed on the surface of the titanium alloy. In addition, the adherent material can be clearly observed.

**Figure 13:**
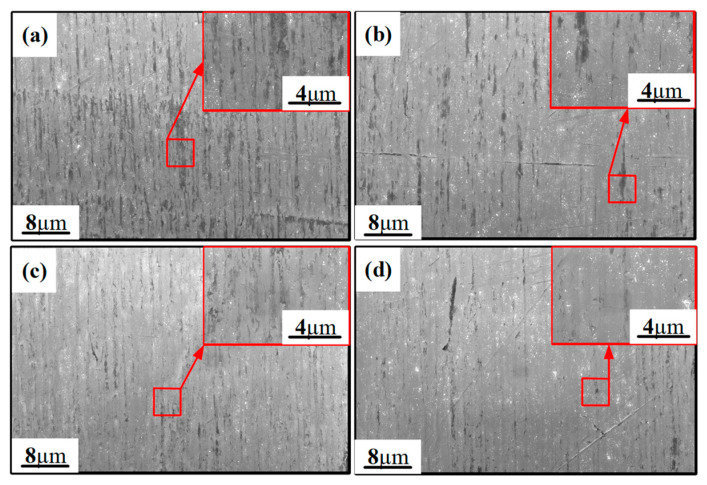
SEM photograph of the surface topography of Ti6Al4V alloy polished by SR and PR bonnet under different dwell times: (**a**) SR bonnet polished under a dwell time of 30 s; (**b**) SR bonnet polished under a dwell time of 300 s; (**c**) PR bonnet polished under a dwell time of 30 s; (**d**) SR bonnet polished under a dwell time of 300 s.

**Figure 14:**
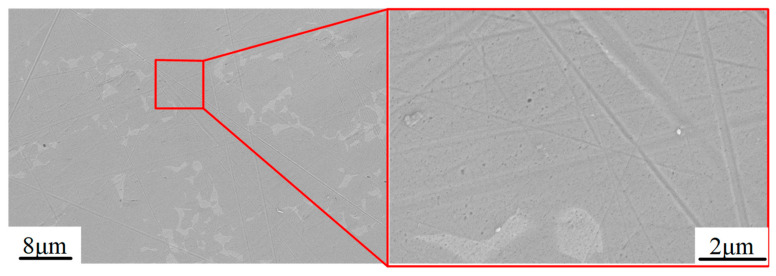
SEM morphology of Ti6Al4V alloy after uniform polishing.

**Figure 15:**
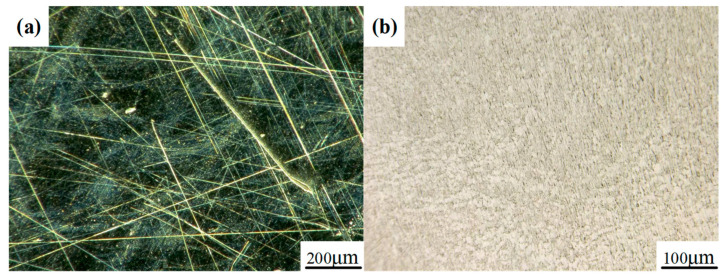
The super depth of field morphology of Ti6Al4V alloy (**a**) before and (**b**) after uniform polishing.

Figure 13 shows the SEM photograph at the center region of the TIF spot under different dwell times. Most of the grinding tool marks were removed and replaced by much smaller abrasive scratches. Specifically, only dense scratches along one direction were observed on the surface when the dwell time was 30 s, as shown in Figure 13a,c. Since the tool was not moving during the generation of the TIF spot, all of the scratches were along the same direction. When the dwell time was 300 s, some deep scratches were found on both spots generated by the SR and PR bonnet, as shown in Figure 13b,d. Although a long dwell time can remove the grinding marks thoroughly, extra deep scratches can be easily generated by some larger abrasives. This phenomenon also explains why the surface roughness increases when the dwell time is longer than a certain time, as shown in Figure 8a,c. Figure 14 shows the SEM topography of the titanium alloy surface after uniform polishing. A highly smooth surface was obtained, and the grinding marks were thoroughly removed. Moreover, much shallower and disorganized scratches were found on the polished surface compared to the results in Figure 13, which indicates that it is effective to use the SR bonnet tool for the rough polishing of Ti6Al4V, followed by the PR bonnet for a finer finish. The super depth of field morphology of Ti6Al4V alloy before and after uniform polishing in Figure 15 also indicates how the grinding tool marks were removed to obtain the ultra-smooth surface.

### 3.5. Surface Material Composition Analysis before and after Polishing

**Figure 16:**
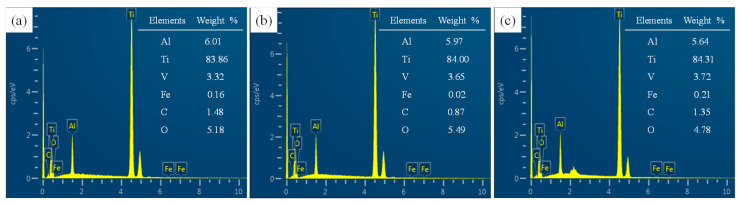
EDX results of typical areas of Ti6Al4V alloy surface before and after polishing: (**a**) EDX results before polishing; (**b**) EDX results of surface after polishing using SR bonnet; (**c**) EDX results of surface after polishing using PR bonnet.

**Figure 17:**
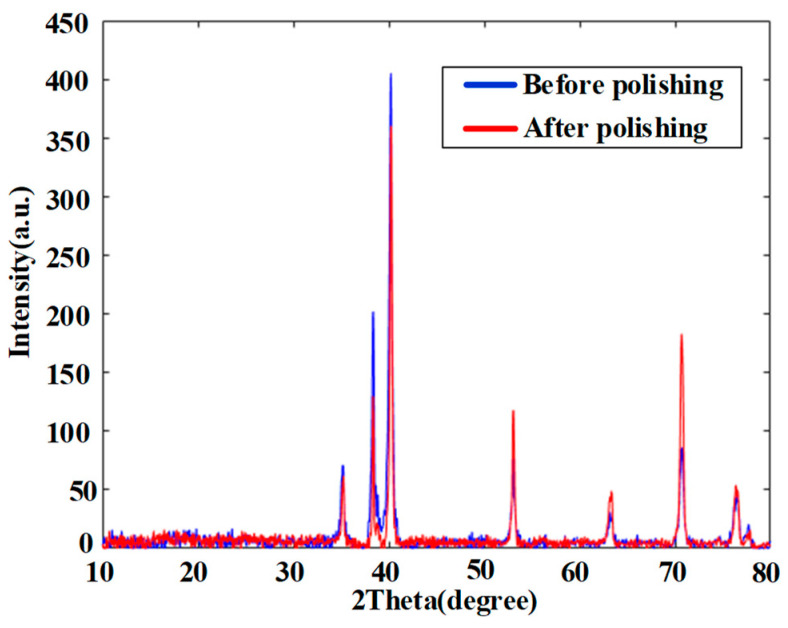
XRD of titanium alloy before polishing and after final polishing using PR bonnet.

To investigate whether chemical reactions or material changes took place before and after polishing, EDX and XRD analysis were carried out. The EDX analysis results are shown in Figure 16, demonstrating that the surface chemical composition of the Ti6Al4V was basically unchanged. This reveals that the adhesion of titanium alloy during polishing is not due to a chemical reaction. At the same time, the phase detection of the titanium alloy before and after final polishing was carried out by XRD. As shown in Figure 17, the XRD curves are quite similar before and after polishing. Hence, no phase change occurred after polishing. Even though there are some variations at certain peaks, these may be due to the differences in surface roughness.

### 3.6. Comparison of SR Bonnet Polishing to Other Polishing Methods

Several different polishing methods have been developed for the polishing of Titanium alloys. Table 5 presents a general comparison between different polishing methods, including laser polishing, belt polishing, abrasive flow machining, chemical mechanical polishing, and SR bonnet polishing. The effects of polishing performance on surface accuracy, polishing efficiency, stability, polishing cost, and adaptability to complicated surfaces are evaluated. However, we must state that some of these effects may not fit this general comparison for specific tools. Generally, the comparison indicates the superior performance of SR bonnet polishing considering these factors. Hence, it has the potential to be used as an attractive polishing tool to enhance the efficiency of titanium polishing.

## 4. Conclusions

In this paper, we studied the material removal characteristics of two bonnet polishing tools with different rigidities for the polishing of Ti6Al4V alloy material. Two kinds of bonnet tool were used, namely a pure rubber (PR) bonnet and a semirigid (SR) bonnet. The characterization and comparison of the material removal and surface integrity before and after polishing were conducted through a series of experiments on a 6-DOF robotic polishing device. Several groups of tool influence functions (TIFs) under different conditions were generated to compare their material removal rate and polished surface roughness. Moreover, uniform polishing experiments on a plane Ti6Al4V surface were also carried out to test the rough polishing and fine polishing performance, as well as the polished surface integrity. The following conclusions can be drawn.

1. Both SR bonnet and PR bonnet can be successfully utilized for the polishing of Ti6Al4V alloy to obtain nanometric surface roughness.
2. The material removal rate of SR bonnet was found to be significantly higher than that of PR bonnet on Ti6Al4V alloy by comparing the volume removal rate of their tool influence functions (TIFs).
3. SR bonnet is suitable for rough polishing to significantly reduce surface roughness, and PR bonnet is suitable for fine polishing. The surface roughness of ground Ti6Al4V alloy was changed from an initial 172.1 nm Sa to a highly smooth surface of 6.1 nm Sa by combining rough polishing and fine polishing using SR bonnet and PR bonnet, respectively.
4. No obvious chemical reaction or phase change was observed during the polishing of Ti6Al4V alloy using SR and PR bonnet according to the EDX and XRD analysis.

## Figures and Tables

**Table 1:** Chemical composition of Ti6Al4V titanium alloy (weight %).

Fe	C	N	H	O	Al	V	Ti
≤0.30	≤0.08	≤0.05	≤0.015	≤0.20	5.5~6.75	3.5~4.5	allowance

**Table 2:** TIF generation conditions.

	Group 1						Group 2			
	1	2	3	4	5	6	1	2	3	4
Dwell time (s)	30	60	120	180	240	300	0.4			
Tool offset (mm)	180						0.2	0.3	0.4	0.5
Others	θ=15°, inner pressure=0.1 Mpa, ω = 1000 rpm									

**Table 3:** Uniform removal conditions.

Conditions	Semirigid Bonnet	Pure Rubber Bonnet		
Tool radius (mm)	40	40		
Polishing times (cycle)	10	2	2	5
Precession angle (deg)	15	15	15	15
Inner pressure (MPa)	0.1	0.1	0.1	0.1
Speed (rpm)	1000	1000	1000	1000
Offset (mm)	0.4	0.4	0.4	0.4
Feed rate (mm/min)	100	100	100	100
Polishing slurry	~3 wt.% alumina, 5 μm in average	~3 wt.% alumina, 2 μm in average	~3 wt.% alumina, 0.5 μm in average	~3 wt.% alumina, 0.05 μm in average

**Table 4:** Section size of polishing point.

Dwell Time(s)	Semirigid (SR) Bonnet		Pure Rubber (PR) Bonnet	
	Diameter (mm)	Depth (μm)	Diameter (mm)	Depth (μm)
30	12.2	1	12	1
60	12.6	1.3	12.2	1.2
120	13.2	1.5	12.6	1.3
180	13.8	2	13.0	1.8
240	14.0	2.7	13.0	2.5
300	14.6	3	13.6	2.8

**Table 5:** General comparison between different tool types ^a,b,c^.

	Laser Polishing [25,31,45]	Belt Polishing [24,30,44]	Abrasive Flow Machining [26,46]	Chemical Mechanical Polishing [28,47,48,49]	**Semirigid** **(SR) Bonnet** [40,41,42,43]
Surface accuracy	Medium	Low	Low	**Excellent**	**Excellent**
Polishing efficiency	**Good**	**Excellent**	Medium	Medium	**Excellent**
Stability	**Excellent**	Medium	Medium	**Excellent**	**Good**
Polishing cost	High	Medium	Low	Medium	**Low**
Adaptability to complicated surfaces	**High**	Medium	Medium	Low	**High**

Note: Bold items are usually regarded as advantages. ^a^ These characteristics are mainly considered when evaluating a polishing tool. ^b^ This is only a general comparison. These characteristics may vary for specific tools. ^c^ Blue color means better performance.

## Data Availability

Not applicable.
